# Effects of flood irrigation on the risk of selected zoonotic pathogens in an arid and semi-arid area in the eastern Kenya

**DOI:** 10.1371/journal.pone.0172626

**Published:** 2017-05-31

**Authors:** Bernard Bett, Mohammed Y. Said, Rosemary Sang, Salome Bukachi, Salome Wanyoike, Shem C. Kifugo, Fredrick Otieno, Enoch Ontiri, Ian Njeru, Johanna Lindahl, Delia Grace

**Affiliations:** 1 International Livestock Research Institute, Nairobi, Kenya; 2 Kenya Medical Research Institute, Nairobi, Kenya; 3 Institute of Anthropology, Gender and African Studies, University of Nairobi, Nairobi, Kenya; 4 Department of Veterinary Services, Ministry of Agriculture, Uthiru, Nairobi, Kenya; 5 Division of Disease Surveillance and Response, Ministry of Public Health and Sanitation, Kenyatta National Hospital, Nairobi, Kenya; 6 Department of Clinical Sciences, Swedish University of Agricultural Sciences, Uppsala, Sweden; 7 Zoonosis Science Center, Dept. of Medical Biochemistry and Microbiology Uppsala University, Uppsala, Sweden; George Mason University, UNITED STATES

## Abstract

To investigate the effects of irrigation on land cover changes and the risk of selected zoonotic pathogens, we carried out a study in irrigated, pastoral and riverine areas in the eastern Kenya. Activities implemented included secondary data analyses to determine land use and land cover (LULC) changes as well as human, livestock and wildlife population trends; entomological surveys to characterize mosquitoes population densities and species distribution by habitat and season; and serological surveys in people to determine the risk of Rift Valley fever virus (RVFV), West Nile fever virus (WNV), dengue fever virus (DFV), *Leptospira* spp. and *Brucella* spp. Results demonstrate a drastic decline in vegetation cover over ≈25 years particularly in the irrigated areas where cropland increased by about 1,400% and non-farm land (under closed trees, open to closed herbaceous vegetation, bushlands and open trees) reduced by 30–100%. The irrigated areas had high densities of *Aedes mcintoshi*, *Culex* spp. and *Mansonia* spp. (important vectors for multiple arboviruses) during the wet and dry season while pastoral areas had high densities of *Ae*. *tricholabis* specifically in the wet season. The seroprevalences of RVFV, WNV and DFV were higher in the irrigated compared to the pastoral areas while those for *Leptospira* spp and *Brucella* spp. were higher in the pastoral compared to the irrigated areas. It is likely that people in the pastoral areas get exposed to *Leptospira* spp by using water fetched from reservoirs that are shared with livestock and wildlife, and to *Brucella* spp. by consuming raw or partially cooked animal-source foods such as milk and meat. This study suggests that irrigation increases the risk of mosquito-borne infections while at the same time providing a protective effect against zoonotic pathogens that thrive in areas with high livestock population densities.

## Introduction

Emerging infectious diseases cause a significant burden in the sub-Saharan Africa as: (i) the prevailing climatic conditions favor the development of a wide range of vectors and pathogens throughout the year, (ii) a large proportion of people derive their livelihoods from activities (e.g. crop farming, livestock husbandry, hunting, etc.) that force them to work in remote and high risk areas, and (iii) access to reliable human and animal health services is a major challenge. This burden is expected to increase as the on-going demographic and climatic changes put more strain on natural and managed ecosystems. The demand for food, for example, is expected to increase with the rising human population and, where possible, more agricultural intensification (e.g. irrigation) will be required to bridge the expected food deficits.

Whereas irrigation enhances agricultural production, it can erode biodiversity and weaken supporting and regulatory ecosystem services as diverse species of animals, plants and ecological communities and functions get replaced by crop monocultures raised for food and fodder production [1]. It also increases the suitability of a habitat for mosquito colonization, potentiating the risk of multiple mosquito-borne infections. Many studies have associated flood irrigation with increased incidence of mosquito-borne diseases such as malaria [2–3]. In other instances though, irrigation has had a protective effect on these diseases as improved socio-economic conditions of the local people associated with improved agricultural productivity has enhanced their capacity to access health services [4]. The magnitude and direction of altered disease incidence due to irrigation and LULC changes in general would therefore depend on the ability of the changes introduced to establish niches for pests and pathogens and the capacity of the local people to protect themselves [5].

This study investigated the effects of flood irrigation on the risk of zoonotic diseases that included mosquito-borne infections, Rift Valley fever virus (RVFV), West Nile virus (WNV) and Dengue fever virus (DFV) and important bacterial zoonoses such as *Brucella* spp and *Leptospira* spp. It used lessons learnt from earlier but similar studies on malaria and schistosomosis to investigate the effects of irrigation on emerging infectious pathogens e.g., RVFV and to determine how this intervention would affect the risk of key bacterial zoonoses, *Brucella* spp. and *Leptospira* spp. that thrive in areas with high livestock/wildlife population densities.

## Material and methods

### Analytical design

The expected linkages between key drivers of change (land use, human population, and climate changes) on peoples’ health and wellbeing were determined using a causal web model. Irrigation was expected to enhance water availability, food and fodder production but at the same time increase the risk of water-associated diseases, increased exposure to agricultural chemicals and reduced access to animal source foods. Key areas of research that this work could focus on were identified as (i) ecological studies to determine trade-offs in ecosystem services, (ii) disease transmission dynamics, and (iii) social and livelihood difference assessments.

### The study areas

**Fig 1:**
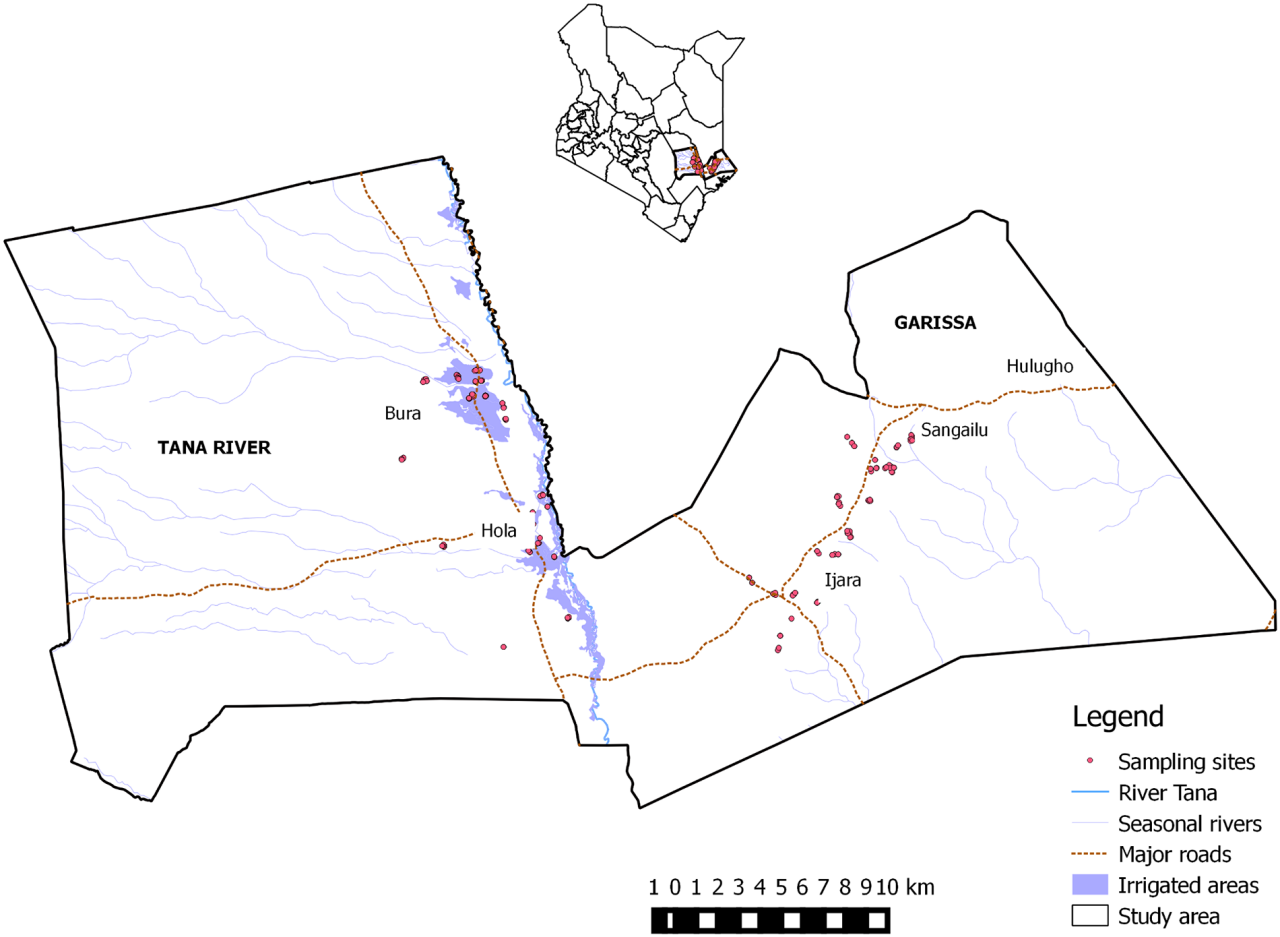
Map of the sampling sites in Tana River and Garissa counties. The inset map shows the location of the study area in Kenya.

The study was conducted in Bura and Hola in Tana River County, and Ijara and Sangailu, Garissa County, both in eastern Kenya (Fig 1). Tana River separated these areas as Bura and Hola were found on the western side of the river while Ijara and Sangailu were on the eastern side. Two commercial irrigation and settlement schemes Hola and Bura, developed in 1953 and 1978 respectively, were purposefully chosen for the study. Ijara and Sangailu in Garissa County, pastoral rangelands, were chosen as the control sites because their migratory corridors for people and livestock rarely reached the irrigation schemes. The study areas could be therefore classified into three distinct ecosystems, namely riverine, pastoral rangeland, and irrigated areas depending on their ecological characteristics and livelihood activities used by the local people.

The riverine area is a biodiversity hotspot that supports unique plant taxonomy and more than 200 birds and 57 mammal species including two highly endangered primates, the red colobus (*Colobus badius rufomitratus*) and the crested mangabey (*Cercocebus galeritus galeritus)* [6]. This vegetation is recognized as an endangered ecosystem, suffering forest losses due to natural river course changes in combination with altered flooding regimes from upstream dams, land conversion for agriculture and irrigation schemes, and extractive forest use [7].

Bura irrigation and settlement scheme was developed to settle landless farmers, boost food and cash crop production, generate employment and provide water in an arid and semi-arid area. It currently has a capacity of 2,100 ha with a tenant population of slightly over 2,000 households settled in 10 villages (https://www.nib.or.ke/projects/public-irrigation-schemes/bura-irrigation-scheme). Hola irrigation and settlement scheme has a total area of 1,011 ha, and at its inception, the scheme had 700 farming households settled in 6 villages (http://softkenya.com/farming/irrigation-in-kenya/). Agricultural activities were interrupted in 1989 when River Tana, the main source of water, changed course at the water intake point. The main crops grown in these schemes include maize and cotton.

Ijara sub-County covers an area of 9,778 km^2^. It borders Lamu County and Boni forest to the East and Tana River County to the West. It lies approximately between latitudes 1° 7`S and 2° 3’S and longitudes 40° 4’E and 41° 32’E. The sub-County has an altitude of 0–90 m above sea level and its annual mean temperature ranges between 15°C–38°C and annual rainfall between 750mm– 1000 mm [8]. The types of soils found in the area are solonetz and vertisols [9]. Pastoralism is the main socio-economic activity with Boran cattle, red Maasai sheep, black head Persian sheep and Galla goats being the predominant livestock breeds kept [8].

The study areas were enclosed within a geographical block represented by the minimum and maximum longitude and minimum and maximum latitude of 38.93°E, 41.14°E, 1.89°S, and 0.64°S, respectively.

### Data collection

#### Secondary data

Secondary data on LULC and human, livestock and wildlife population trends were collated and used to characterize the study areas. LULC data were obtained from Africover Multipurpose Land Cover Database for Kenya supported by the United Nation’s Food and Agriculture Organization (FAO) [10]. These data were mapped out in QGIS v2.2 –Valmiera [11]. Changes in LULC between 1975, just before the development of the Bura irrigation scheme, and the year 2000 was analyzed and results tabulated. The Land Cover Classification System (LCCS) described by FAO [10], which provides a comprehensive and standardized classification system, was used to generate the land cover legend.

Data on human population densities were obtained from the National Bureau of Statistics, while livestock and wildlife data were obtained from the Department of Resource Surveys and Remote Sensing (DRSRS), Ministry of Environment, Nairobi. DRSRS has been monitoring livestock and wildlife populations in Kenya’s rangelands using aerial sample surveys since 1977. The survey flights follow systematic, parallel east-west flight lines or transects spaced at 5 km [12]. Findings from these analyses are given in the on-line supporting document.

#### Field surveys

Field surveys included focus group discussions, mosquito sampling and seroprevalence surveys in people.

Focus group discussions (FGDs) based on the techniques described by Bett et al. [13], Jost et al. [14] and others were used to ground-truth the secondary data. They helped to verify types of livestock species kept and their relative distribution and contact with wildlife. The results from these surveys were integrated with those from the secondary data analysis to classify the study areas into at least three ecologies with perceived biodiversity gradient from high, moderate to low.

Mosquito sampling was done in four repeated cross-sectional surveys that were implemented over a period of one year in all the three areas. The first survey was done during the dry period when the level of irrigation was low (i.e. no commercial farming was being done and irrigation was being used only in the gardens). The second trip was done in the dry period when irrigation was high (when commercial maize farming was on-going), the third was during the wet season when commercial farming under irrigation was being done, while the last survey was done during the wet season when there was no irrigation.

Mosquitoes were trapped using CO_2_-baited CDC light traps (John W. Hock, Gainesville, FL) at various points within the three study areas. The specific sampling sites included homesteads, animal enclosures and farms. Traps were set at 4:00pm and left overnight until 6:00am.Trapped mosquitoes were collected every morning and transferred to a field laboratory where they were immobilized using 99.5% triethyleamine (Sigma-Aldrich, St. Louis, MO) and sorted. They were then preserved in liquid nitrogen and transported to the Kenya Medical Research Institute (KEMRI) laboratory for identification.

Mosquitoes were identified to species level using the available taxonomic keys appropriate for identifying all African species including Edwards [15], Gillies and de Meillon [16], Jupp [17], Gillies and Coetzee [18] and Harbach [19]. All identifications were done on ice packs. Identified mosquitoes were pooled in groups of up to 25 and preserved at -80°C for further analyses. A detailed description of the methods used for entomological work is given by Sang et al. [20].

Cross-sectional surveys were implemented to determine seroprevalences of the selected zoonotic pathogens in people. Seropositivity was assumed to represent the risk of exposure to each of the pathogens considered. A person and household represented primary and secondary units of analysis, respectively.

Methods for determining the required sample sizes for comparing independent proportions using a two-sided test described by Dohoo et al. [21] were used for estimating the required sample size. A *priori* seroprevalence of at least one pathogen in irrigated and pastoral study areas, *p*_1_ and *p*_0_, were assumed to be 10% and 5%, respectively. Other assumptions were (i) the level of confidence on the difference between these proportions was 95%; and (ii) the power of the study to find a difference in the prevalences was 80 per cent. Given that people from the same households would have correlated measurements, the estimate produced was adjusted for the design effect assuming an intra-household correlation coefficient of 0.04. This analysis suggested that the study needed to use 550 subjects per area. The number of households required (n = 110 per area) was determined by assuming that up to five subjects per household would be sampled. Only pastoral and irrigated areas were considered at the design stage of the study; the riverine area was included at the data collection stage of the project when it was realized that some of the households sampled lived in a riverine area.

Households were identified using random selection based on a sampling frame that was developed during recognizance visits. Blood samples were obtained from at least five members of selected households using aseptic procedures by qualified phlebotomists from the Ministry of Health. Up to 20 ml venous blood were obtained from patients above 10 years and 15 ml from those between 5–10 years using sterilized butterfly needles and vacutainer tubes. Half of the sample was collected in non-heparinised tubes for serum preparation and serological analysis while the other was collected in herparinised tubes. Serum samples obtained were stored and transported to the Kenya Medical Research Institute (KEMRI) laboratories in dry ice for serological testing using the available and validated enzyme-linked immunosorbent assays (ELISA). Table 1 outlines the types of ELISA kits used for each pathogen.

**Table 1:** Names and producers of the ELISA kits used for serological tests conducted.

Pathogen	Kit name	Manufacturer
Rift Valley fever virus	RVF Inhibition ELISA	BDSL, National Institute for Communicable Diseases, Centre for Emerging and Zoonotic Diseases, Johannesburg, South Africa
West Nile fever virus	West Nile Detect ^™^ IgG ELISA	InBios International, Inc., Seattle, WA., USA.
Dengue fever virus	DENV Detect ^™^ IgG ELISA	InBios International, Inc., Seattle, WA., USA.
*Brucella* spp.	Brucella abortus IgG ELISA	Demeditec Diagnostics GmbH, Kiel, Germany.
*Leptospira* spp.	Panbio^®^ Leptospira IgM, ELISA	Panbio Diagnostics, Standard Diagnostics, Inc., Alere ^™^, Australia.

### Data analysis

#### Livestock and wildlife population trends

The analysis of livestock and wildlife population estimates followed the methods described by Ogutu et al. [22]. Briefly, estimates were averaged by species for the periods 1970s, 1980s, 1990s and 2000s to minimize stochastic variation on the estimated population sizes due to sampling. Complete data were available only for the Tana River County and so the findings obtained only relate to the irrigated sites. The population estimates and standard errors were based on Jolly II method [23] and the differences between years or period were based on z-statistics [12].

#### Analysis of mosquito population density and seroprevalence data

Descriptive analyses were done to show the distribution of the apparent mosquito population densities and seroprevalence of the target pathogens across the three areas. Thereafter, statistical analysis was done using a Bayesian geostatistical model with appropriate link functions (normal and binomial link functions for the mosquito and seroprevalence data, respectively) and predictor variables. The model was formulated using the RINLA algorithm proposed by Rue et al. [24]; it was of the form

$$\eta_{i}=\beta_{0}+\sum_{m=1}^{M}\beta_{m}x_{mi}+f(z_{i})$$ (1)

Where

*η*_*i*_ is the linear predictor linked to the original scale of the outcome *y*_*i*_ through a link function, *β*_0_ is a scalar representing the intercept, *β*_*m*_ represent the values of the coefficients quantifying the linear effect of covariates *x*_*m*_, and *f*(*z*_*i*_) is a function used to account for the spatial random effect. For mosquito data, *y*_*i*_ was a log-transformed number of mosquitoes captured in a trap per day, *m*, with 1 being added to this number to ensure that records with no catches did not return errors i.e. *log*(*m*+1). For the seroprevalence data, *y*_*i*_ was a binary variable, 1 representing a positive test result and 0 otherwise.

Predictors considered for the mosquito data included rainfall, temperature, humidity, area, and irrigation intensity (low, moderate and high). For the seroprevalence data, area was the only variable used to determine if there were significant variations in the distribution of the pathogens by area. A spatial random effect implemented using stochastic partial differential equations (SPDE) was used to account for spatial autocorrelations and unmeasured factors in these areas. The spatial domain was defined using the shape file of the study area obtained from ILRI’s GIS database. The significance of the independent factors was assessed using credible (5–95%) intervals generated as part of the posterior distributions of the model parameters. The significance of the spatial effect was determined using deviance information criterion (DIC) statistic. For this analysis, two hierarchical models—with and without the spatial effect—were fitted to the data and the model that provided a smaller DIC estimate was preferred.

#### Ethics

The ethical approval for the study was obtained from the African Medical Research Foundation (AMREF) Ethics Committee and each subject selected for sampling signed a written consent before being recruited. Approvals required for sampling in Sangailu and Ijara were obtained from the Ministry of Health Garissa County while those for Bura and Hola were provided by both the Ministry of Health, Tana River County and the representatives of the National Irrigation Board in each of these locations.

## Results

### LULC changes

The pastoral area had more very open trees (40–15% crown cover), trees and shrub savannah and closed trees compared to the irrigated area (Table 2). There was also more area under urban and rural settlements in the irrigated compared to the pastoral area. As expected, irrigated herbaceous crop, water bodies and rice fields were found only in the irrigated/Tana River area.

**Table 2:** Areas (in %) covered by selected land use land cover types in Tana River, an area with extensive land use changes, and Ijara, the pastoral site with limited land use changes (control site).

Land use land cover types	Area (%)	
	Tana River/irrigated	Ijara/pastoral
Very open trees (40–15% crown cover)	46.88	72.41
Very open shrubs (40–15% crown cover)	22.26	0.40
Open to closed herbaceous vegetation	10.3	6.99
Open trees on temporarily flooded land	4.26	1.15
Open to closed herbaceous vegetation on temporarily flooded	3.56	0.13
Trees and shrubs savannah	1.38	5.13
Closed trees	0.91	4.24
Irrigated herbaceous crop	0.78	0.00
Water bodies	0.57	0.00
Rice fields	0.41	0.00
Urban and associated areas, rural settlements	0.15	0.07

The analysis on LULC changes focused on the irrigated areas in Tana River County for the period 1975 to 2000; this showed that a number of key habitats had been lost over the period including closed tree, open to closed herbaceous vegetation, bushlands, and open trees (Fig A in S1 File). A large increase in land cover was in cropland/irrigation where there was an increase of more than 1,400%, followed by open tress on temporally flooded land (50%) and herbaceous vegetation on flooded land (48%). Open shrubs and sparse shrubs had about 10% increase in area.

### Livestock, wildlife and human population trends

Analyses on the trends of livestock, wildlife and human populations between the 1970s and 2000s show that there were no significant changes in the populations of cattle (n = 45,000; P = 0.33) and donkeys (n<5,000; p = 0.49), but those for shoats (sheep and goats) rose substantially in the 2000s. The decline of buffalo, elephant, Burchell’s zebra and impala has been precipitous and these animals were not observed in censuses conducted in the 2000’s. Human population has been rising more in Tana River (the irrigated areas) than the neighboring pastoral areas although in the latter, a major change in the population occurred between 1999 and 2009 (Fig B in S1 File).

### Focus group discussions

A total of 42 focus group discussions (FGDs) involving 411 people (194 women and 217 men) were conducted throughout the study areas. These surveys were implemented at the village level and participants were invited to join the discussions irrespective of whether their households had been selected for blood sampling. Fourteen FGDs were held in irrigated areas, 12 in riverine areas and 16 in pastoral areas. The numbers of participants involved were 162 (70 women and 92 men) in irrigated areas in Bura and Hola, 108 (51 women and 57 men) in riverine areas and 141 (73 women and 68 men) in pastoral areas in Ijara and Sangailu divisions. These discussions showed that farmers in the irrigated areas mainly kept goats and sheep with very few cattle while those in the pastoral and riverine areas kept large populations of cattle, sheep and goats. There were no camels in Ijara but a few were in Tana River.

Participatory maps developed in these surveys defined common vegetation types, land use patterns and ecosystem services that communities derived from these areas. The irrigated areas mainly had crop farms with maize, cotton, rice and various horticultural crops, the riverine area had small scale farms interspersed within forested habitats while the pastoral area had savannah grasslands with a few conservancies. People in the irrigated areas identified birds and baboons as the common wild animals in the area while those from riverine and pastoral areas listed a range of wildlife including crocodiles, mongooses, bat-eared foxes, caracal, several rat species, vervet and Syke’s monkeys and hippos.

### Mosquito population densities

**Fig 2:**
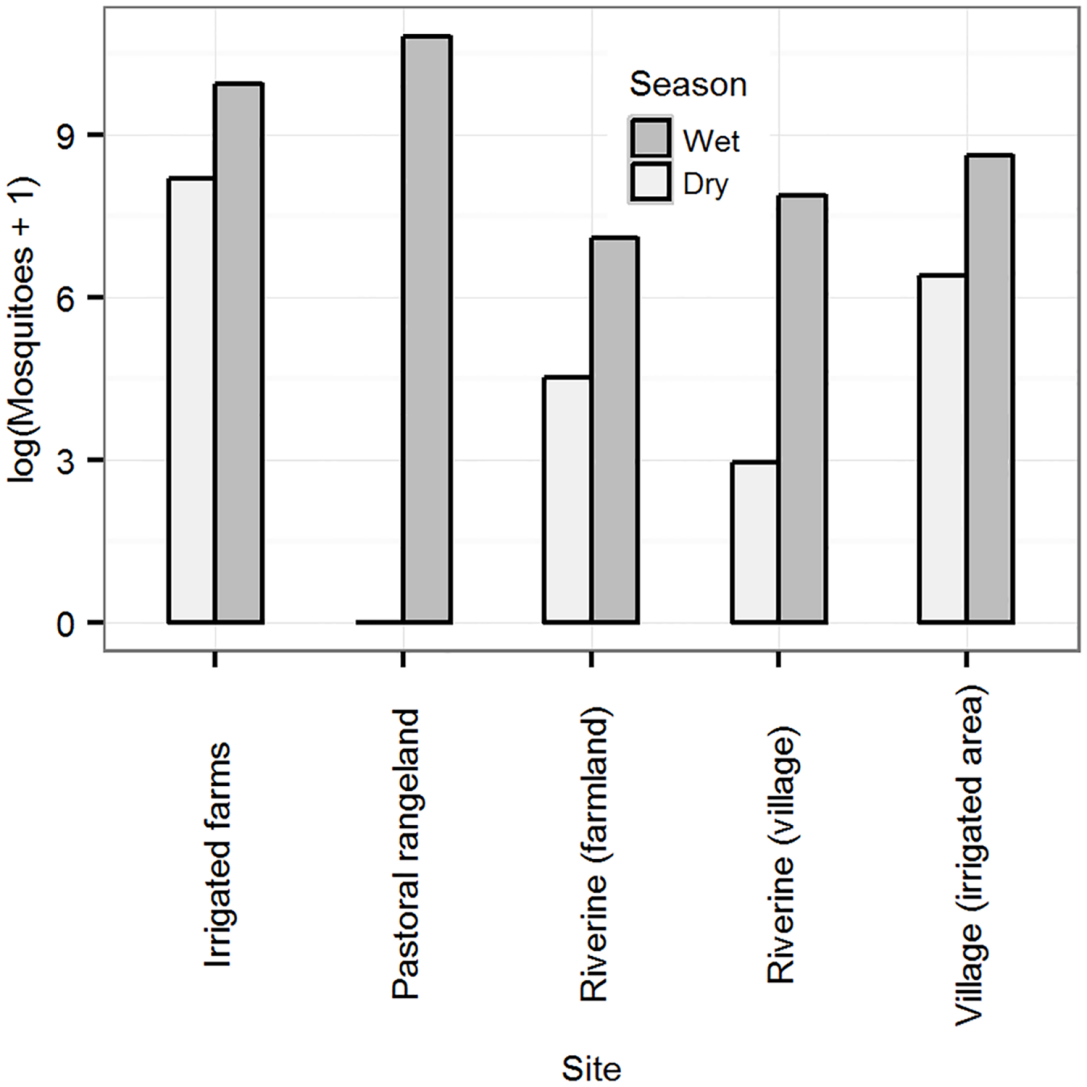
Distribution of mosquito population densities (log transformed) by site and season. Pastoral, Irrigated farms, Village (irrigated area), Riverine-farmland, Riverine-village.

A higher proportion of mosquitoes (94.8%, n = 79,725) were trapped in the wet than the dry season and most of these were from the pastoral areas. Of the mosquitoes collected in the dry season, 83.5% (n = 3,592) were from farms in the irrigated area. There was a bigger variation in densities of mosquitoes between areas in the dry season, with levels in the pastoral areas being lower than the detection level while the irrigated farms had relatively higher densities compared to the other two areas (Fig 2).

Mosquito species that were found mainly in irrigated farms compared to the other areas included *Aedes* (*Ae*.*) mcintoshi*, *Ae*. *tricholabis*, *Aedomyia furfurea*, *Culex (Cx*.*) bitaeniorhynchus*, *Cx*. *univittatus*, *Mansonia (Mn*.*) africana*, *Mn*. *uniformis* and those that were found more in the villages or settlement areas compared to the irrigated areas were *Ae*. *aegypti*, *An*. *gambiae*, *Anopheles (An)*. *squamosus* and *An*. *funestus*. A large percentage (65.8%, n = 1,677) of the mosquitoes trapped in the pastoral area was *Ae tricholabis*. Others were *Ae*. *ochraceus*, *Ae*. *mcintoshi* and *Ae*. *sudanensis*.

The model fitted to these data identified area/LULC, season and humidity as significant predictors. Compared to the irrigated farms, all the areas sampled, i.e., riverine farm, riverine village, irrigation village and pastoral area had lower mean effect on the mosquito population in that descending order (Table 3). Specifically, the pastoral rangeland had the lowest relative mean effect which was also significantly different from that of the irrigated farms. Wet season had a positive effect compared to the dry season and humidity was also positively correlated with mosquito population density. The model with spatial random effect fitted the data better than the one without based on the DIC estimates generated.

**Table 3:** Outputs of a geostatistical model illustrating the effects of land use, season and humidity on mosquito population densities. The regression parameters are mean and percentile ranges (2.5–97.5%) of posterior distributions of fixed and random effects.

Variable	Levels	Mean	Percentile Range	
			2.5%	97.5%
Site/LULC	Farm—riverine area	-0.16	-1.08	0.77
	Village—riverine area	-0.45	-1.25	0.34
	Village—irrigation scheme	-0.86	-1.19	-0.53
	Pastoral rangeland	-2.27	-2.99	-1.55
	Irrigated farm	0.00		
Season	Very wet	1.84	1.23	2.46
	Wet	0.20	-0.17	0.57
	Dry	0.00		
Humidity		0.03	0.03	0.04
Model hyperparameters:				
Precision for the Gaussian		1.23	1.01	1.48
*θ*_*1*_		-6.30	-8.89	-3.99
*θ*_*2*_		4.42	3.06	5.92

DIC estimates for models with and without spatial effect: 702.50 verses 726.9

### Seroprevalence of the selected zoonotic pathogens in people

The seroprevalences of RVFV and combined WNV/DFV were marginally higher in the riverine area compared to irrigated and pastoral areas while those of *Brucella* spp. and *Leptospira* spp. were always higher in the pastoral areas (Table 4). These subjects were sampled from 15, 23 and 3 villages in irrigated, non-irrigated and riverine areas, respectively. The multivariable models fitted to the data suggest that the seroprevalence of RVFV was higher in riverine areas compared to the pastoral rangeland, while that for WNV/DFV was higher in riverine and irrigated area compared to the pastoral rangeland (Table 5). These differences were however not statistically significant. Seroprevalences for *Brucella* spp and *Leptospiral* spp had an opposite pattern. The parameters of the regression model (Table 5) show that the pastoral area had relatively higher risk of exposure to these pathogens compared to the riverine and irrigated areas.

**Table 4:** Descriptive analysis of the seroprevalence data illustrating frequency distribution of the human subjects sampled and prevalences of RVFV, WNV/DFV, *Brucella* spp and *Leptospira* spp by study site.

Area	Pathogen											
	RVFV			WNV/DFV ^a^			*Brucella* spp			*Leptospira* spp		
	n	p ^b^ (%)	SE	n	p ^b^ (%)	SE	n	p ^b^ (%)	SE	n	p ^b^ (%)	SE
Irrigation scheme	303	21.12	0.023	88	28.91	0.048	293	16.38	0.022	252	15.08	0.022
Riverine area	81	27.16	0.049	78	33.33	0.053	72	11.11	0.037	71	19.72	0.047
Pastoral area	728	21.70	0.015	496	15.73	0.016	652	47.55	0.020	625	30.72	0.018

^a^ WNV and DFV data are combined because no confirmatory tests for the ELISA results have been done and their trends across the areas were similar

^b^ Seroprevalence

**Table 5:** Outputs of a geostatistical model illustrating the association between land use and seroprevalence of RVFV, WNV/DFV, *Brucella* spp and *Leptospira* spp. The regression parameters are mean and percentile ranges (2.5–97.5%) of posterior distributions of fixed and random effects.

Variable	Pathogen											
	RVFV			WNV/DFV^a^			*Brucella* spp			*Leptospira* spp		
	Mean	Percentile range		Mean	Percentile range		Mean	Percentile range		Mean	Percentile range	
		2.5%	97.5%		2.5%	97.5%		2.5%	97.5%		2.5%	97.5%
Fixed effects												
Irrigation scheme	0.29	-0.34	0.94	0.19	-0.62	0.91	-1.47	-2.30	-0.65	-0.74	-1.61	0.16
Riverine area	0.12	-0.70	0.92	0.41	-0.44	1.17	-2.25	-3.43	-1.06	-0.90	-1.99	0.22
Pastoral area	0.00			0.00			0.00			0.00		
Random effects—SPDE2 model												
*θ*_*1*_	-2.12	-3.12	-1.09	-1.29	-2.74	0.17	-4.45	-5.84	-3.02	-3.44	-4.42	-2.46
*θ*_*2*_	0.68	-0.40	1.69	0.03	-1.06	1.05	3.25	2.14	4.34	2.20	1.20	3.20

^a^ WNV and DFV seroprevalences are combined as mentioned under Table 4

## Discussion

This study investigated the effects of flood irrigation in eastern Kenya on seroprevalences of RVFV, WNV/DFV, *Brucella* spp and *Leptospira* spp. The introduction of irrigation in the area precipitated considerable socio-ecological changes that resulted in the replacement of rangelands with crops, reduction in livestock and wildlife and alteration of peoples’ livelihoods. Such man-made ecological transformations have occurred in many areas over the last 50 years, with the prominent changes being those linked to water development projects [25]. Many studies have demonstrated how these development projects increase the risk of vector-borne diseases by enhancing vector population densities. On the contrary, not much has been done to show the protective effects of irrigation on other infectious pathogens.

The analyses presented enabled the classification of the study areas into three successive levels of ecological diversity in terms of vegetation cover and mammalian host range classified as low, moderate and high. The irrigated areas were considered as having low biodiversity given that it had limited range of habitats and livestock and wildlife species. Sheep and goats comprised the largest proportion of the livestock species kept. However, the drainage canals and food crops grown in the area attracted birds, baboons and some wild animals especially during the dry periods. The pastoral area were deemed to have moderate levels of biodiversity since it had had many livestock, wild animals and birds. The pastoral area in Ijara were adjacent to the Boni forest which was commonly used as the dry season grazing zone. Analyses on the LULC patterns also show that Ijara had more woody vegetation cover than the irrigated areas in Tana River. The riverine area had relatively higher biodiversity compared to the irrigated area. It had many wild animals and birds and a good vegetation cover. The woodland vegetation in the area provided vital ecosystem services such as trapping and regulating river flow and was used by the local people as a source of construction materials and medicinal products. The area also had remnant riverine forests and was among the most important habitats in Kenya for biodiversity conservation [7].

The distribution of the zoonotic pathogens studied varied by area. The mosquito-borne pathogens, RVFV and WNV/DFV were more prevalent in irrigated and riverine areas than the pastoral areas, while bacterial pathogens, *Brucella* and *Leptospira* spp. were prevalent in the pastoral areas. These patterns can be explained based on the prerequisites for pathogen transmission described by Lambin et al. [26] which suggest that pathogen transmission depends on the presence of i) animal donors; ii) vectors; iii) animal recipients; iv) the pathogen in an infective state, and v) factors that influence the external environments contributing to an unhindered transmission of infection from one host to another.

The irrigated and riverine areas had good conditions for the development of mosquitoes throughout dry and wet seasons. The risks of RVFV and WNV/DFV were therefore higher in these compared to the pastoral areas. In both irrigated and riverine areas, there were higher apparent densities of *Ae*. *mcintoshi*, a vector that is thought to maintain RVFV trans-ovarially. Livestock and wildlife hosts that were present in the irrigated and non-irrigated areas were expected to sustain endemic transmission of the virus with occasional zoonotic spill-overs as demonstrated by the relatively high seroprevalence of RVFV in people in these areas. Many studies have been done to identify wild reservoir hosts for RVFV but none has provided conclusive findings despite the prevalence of RVFV antibodies in multiple wildlife species [27] [28] [29].

The higher risk of WNV/DFV in the riverine area (which as stated above has more than 200 species of birds and some of the endangered primates) suggests that greater transmissions of these viruses occur in areas with a higher density of reservoir hosts—wild birds. Studies conducted in Louisiana suggested that a high diversity of non-passeriform birds plays a role in dampening WNV amplification in mosquitoes [30] but studies conducted as part of this project in the study areas found out that bird species was not associated with WNV exposure [31]. Our finding are in line with the observations by Keesing et al. [32] which indicates that high host diversity increases the total feeding opportunities for vectors, and hence their survival rates, therefore enhancing encounter rates between the vector and the most competent host.

*Leptospira* and *Brucella* spp. had similar risk patterns but factors that influenced their distribution might be slightly different. Leptospirosis has been regarded as a neglected zoonotic disease but latest reviews and reports consider it a re-emerging disease. It is endemic in swampy areas where the leptospires can survive longer in water and wet soil. Rodents are believed to be the most important maintenance hosts but a wide range of mammals including dogs, cattle, pigs and sheep can also act as hosts for leptospires [33]. Though it is expected that the irrigated area would have higher risk of leptospirosis compared to the pastoral areas, our findings showed a contrary relationship. In the pastoral areas used in this study, people, livestock and wild animals, including a variety of rodents, share few watering points (often open water pans made to trap rain water), which are expected to support intense contact and hence spread of leptospires between hosts. In addition, access to clean drinking water in the irrigated areas might reduce the risk of human exposure. *Brucella* spp. was also found to be more prevalent in the pastoral than the irrigated area. This pathogen is mainly transmitted from livestock to people through the consumption of raw or undercooked milk and meat, which form the highest proportion of diet, especially in children and women in these areas compared to riverine or irrigated areas where grains and other vegetables are consumed. This pathogen is also likely to be insensitive to host diversity. The species that infects goats and camels is more virulent in man than that which infects cattle.

The study used an analytical design which allowed comparisons to be made between irrigated and non-irrigated areas. It would have been more appropriate to use a pre- and post-exposure (irrigation) design or a paired comparisons involving multiple sites [34]. To alleviate some of the weaknesses, the study used two irrigation schemes, multiple villages by area, and multiple diseases in a bid to increase the diversity of the study population. In addition, a Bayesian random effects model that accounted for spatial autocorrelations was also fitted to the data to ensure independence between observations and control for unmeasured factors. One major weakness of the study though was on the use of serological tests to infer risk of exposure to the selected zoonotic pathogens. This might have biased the reported effects of area to the null because antibodies (especially IgG) persist in the circulation for longer periods and make it more difficult to demonstrate recent infections. A longitudinal design that assesses the incidence of pathogen exposure would be required to accurately determine area-specific infectious disease challenge. The other weakness of the study relates with the use of CDC light traps to sample all the species of mosquitoes. Biogents traps are superior for capturing *Aedes* mosquitoes and hence the population density estimates obtained could be conservative. Nonetheless the results obtained indicate that flood irrigation can support the development of a wide range of mosquitoes including the primary vectors of RVF, *Ae*. *mcintoshi*, and indirectly limit the transmission of zoonotic bacteria that thrive well in areas with high livestock populations.

## Conclusions

The ecological analyses conducted demonstrate substantial trade-offs in ecosystem services as areas that have been put under irrigation have had extensive habitat degradation leading to remarkable losses of land cover and host diversity to the benefit of increases in food production. These areas have also had more water as demonstrated by high suitability for colonization by a broad range of mosquitoes.

The epidemiological studies conducted indicate that these land use/land cover changes affect the distribution of the zoonotic diseases studied. As expected, the seroprevalence of mosquito-borne diseases were higher in the irrigated and riverine areas while the risk of *Leptospira* spp. and *Brucella* spp. were higher in the pastoral areas given that peoples’ livelihoods required them to use common environments with livestock and wildlife. The pastoralists also relied on milk and meat from livestock, hence there were more opportunities for exposure to *Brucella* spp compared to those from the irrigated areas where farm products including vegetable comprised the main source of household diet.

## Supporting information

S1 File This is the online supporting file. (DOCX)
