# Supplementary material for: Effects of flood irrigation on the risk of selected zoonotic pathogens in an arid and semi-arid area in the eastern Kenya
Source: PLoS One. 2017 May 31;12(5):e0172626. doi: 10.1371/journal.pone.0172626 (PMC5450996; doi:10.1371/journal.pone.0172626)
Supplement: S1 File — (DOCX) [file pone.0172626.s001.docx]

**S1 Text. Online Supporting Text**

**Effects of flood irrigation on the risk of selected zoonotic pathogens in an arid and semi-arid area in the northeastern Kenya**

Bernard Bett^1^^[[1]](#footnote-1)^, Mohammed Y Said^1^, Rosemary Sang^2^, Salome Bukachi^3^, Salome Wanyoike^4^, Shem C Kifugo^1^, Fredrick Otieno^1^, Enoch Ontiri^1^, Ian Njeru^5^, Johanna Lindahl^1,6,7^ and Delia Grace^1^

^1^International Livestock Research Institute, P.O. Box 30709-00100, Nairobi, Kenya

^2^Kenya Medical Research Institute, P.O. Box 54840-00200, Mbagathi Way, Nairobi, Kenya

^3^Institute of Anthropology, Gender and African Studies, University of Nairobi, P.O. Box 30197-00100, Nairobi, Kenya

^4^Department of Veterinary Services, Ministry of Agriculture; P.O. Box 319-00605, Uthiru, Nairobi, Kenya

^5^ Division of Disease Surveillance and Response, Ministry of Public Health and Sanitation, Kenyatta National Hospital, Kenya

^6^Department of Clinical Sciences, Swedish University of Agricultural Sciences, PO Box 7054, SE-750 07 Uppsala, Sweden

^7^Zoonosis Science Center, Dept. of Medical Biochemistry and Microbiology Uppsala University Box 582, SE-751 23 Uppsala, Sweden

**SI Materials and methods**

**Focus group discussions**

A total of 14 focus group discussions (FGDs) were held in irrigated sites, 12 in riverine sites and 16 in pastoral sites. The check list used covered livelihood strategies, gender, livestock herd dynamics, ecosystem services and diseases. The checklist is given below.

1. **Livelihood issues**
2. Aims of wealth/capital ranking/household diversity profiling:

- To investigate perceptions of wealth inequalities in a village
- To identify and understand local indicators and criteria of wealth and well-being
- To map the relative number of households in a village in relation to the clusters identified.

Questions

- Describe the different types of households found in your village (wealthy/rich, poor etc.)
- List the predominant characteristics of each of the different types of households in your village

1. Type of house structure, number of rooms, roofing materials;
2. Size and/or amount of land farmed;
3. Sources of energy (i.e. firewood, kerosene, solar);
4. Personal and household belongings (e.g. mobile phones, bicycles, tools);
5. Type and number of livestock;
6. Skills and education levels of both parents and children;
7. Livelihood activities;
8. Food security levels

- Describe the nature of land ownership (subdivision, community owned etc.)
- Describe the features of a person who is absolutely comfortable (wellbeing and the characteristics)

1. Main means of Livelihood/Seasonality/gender dynamics

- To identify the common economic activities in a village.
- To identify and understand the seasonal changes in the livelihood activities.
- To establish the gender dimensions in the livelihood activities.

Questions

- List the main means of livelihood in the community (by gender, social & socio-economic status, religion) (Ranking of most common)
- List all the activities related to the main means of livelihood in the community **(livestock:** herding, watering, milking, treating, slaughtering, assisting in the birthing process, preparation and consumption of animal and animal products, caring for diseased animals etc. **Crop farming:** Land preparation, planting, weeding, harvesting, post-harvest activities e.g. drying etc.).
- Gender roles and responsibilities in different forms of activities identified in the different livelihoods.
- Seasonal variations in main livelihood activities by socio-demographic characteristics related to livelihood activities *(Seasonal Calendars; probe for crop farming and livestock keeping)*

1. **Herd Dynamics**

- To establish the livestock movement patterns, population dynamics

Questions

- List all the type of livestock kept (uses of livestock)
- List the different age categories of animals and the local names given to them (between which ages categories is considered a calf, adult etc.)
- Estimate proportions of each age category in a herd
- Estimate proportion of time/years spent in each age category.
- Estimate length of time the animals can be held in a herd.
- Establish the calving seasons
- Establish the number of herds per farmer (where the livestock are normally kept)
- Establish whether animals in different herds meet and mix (how do they mix, when, for how long)
- Map out livestock movement/grazing patterns/routes; where and in which environments such as forested, swampy or grassland areas, seasonal variations, which categories of people are involved in this.
- Establish the livestock markets (Probe: where acquired from and where sold)

1. **Ecosystem**

**Provisioning services/regulating services/cultural services**

- To characterize the existing ecosystem
- To identify the benefits of each ecosystem, the changes and drivers of these change.

Questions

- Using the pictures of the different ecosystems, let the participants identify the pictures that best represent their ecosystem
- List all the services (provisioning, regulating and cultural) in the ecosystems representing the village
- Changes in land use over the years: Probe how has their village/ecosystem changed over the years as far back as they can remember. Probe for changes in the ecosystems services, diseases, pests etc. (historical timelines, community mapping/profiling).
- Establish the drivers/factors responsible for the changes

1. **Diseases/health issues**

- To Identify the problems/diseases associated with each of the ecosystems identified and the variations over time, annually and seasonally
- To establish community linkage/connection of human diseases to animals

Questions

- Common causes of deaths in livestock (causes, seasons with emphasis on diseases)
- Common animal diseases in the ecosystems identified (use of visual aids listing and ranking/incidence scoring/proportional piling/impact matrix scoring).
- Common human diseases in the ecosystems identified (use of visual aids: listing and ranking/incidence scoring/proportional piling/impact matrix scoring).
- Probe how these diseases came about and what causes these diseases and how they vary over seasons and over the years (Probe for both human and animals: seasonal /historical timelines and trends).
- Category of animals usually affected by disease in the herd (Probe: which category and which diseases/conditions)
- Knowledge of diseases that affect both humans and animals (Listing/ proportional piling/incidence scoring).

1. **Rift Valley Fever**

Knowledge and Perceptions regarding RVF:

- Describe RVF: local names and meanings, causes, when does the disease occur, risk factors, symptoms in animals and humans, breeds of animals most affected, categories of people most affected, frequency of occurrence.
- Describe how RVF is controlled and treated in the community for both animals and humans: methods known, methods used, and preferred methods.
- Describe the reporting to relevant authorities of RVF risk: to whom is it done, why, what changes or issues signify RVF risk in the community, how is the authorities response perceived.

Factors associated with transmission and spread of RVF

- Risk factors for the occurrence, transmission and spread of RVF: any specific behaviour that predispose humans to acquiring RVF.
- Describe the perceived linkage of livestock practices (herding, milking, and consumption of animal products, residing with animals) with RVF.
- Describe the perceived linkage of the ecosystem with RVF (show photographs of the different ecosystems such as forests, flooded areas, and grasslands).

Impact of RVF on individuals, families and community

- In what ways does RVF affect individuals, families and communities? {Probe; ability to meet basic needs, impact of illness or death on families, community’s response to such calamities, Government’s response in regard to human and animal health, long term impacts to families and community
- What measures would you like to see in place regarding the control of RVF?

**SI Results**

**Changes in the land use land cover**

A comparison of the land use/land cover analysis for the years 1975 and 2010 showed that more land had been put under cultivation in 2010 (Figure S1).

**Fig. A**. Land cover changes in Tana between 1975 and 2010

**Changes in human, livestock and wildlife populations**

Ijara has three national reserves and one community conservancy. Wildlife species present in the district include the rare Hirola antelope, lions, elephants, buffaloes, monkeys, hippos, crocodiles, guinea fowls, giraffes, ostriches, leopards, hyenas, warthogs, zebra, cheetahs, snakes, deer and varieties of birds. There is one non-gazetted forest. Poaching control measures incorporate routine patrols and participatory wildlife management.

**Fig. B**. Trends of livestock and wildlife in 1970s, 1980s, 1990s and 2000s (a-d) and human population from 1969 – 2009 (e). Livestock and wildlife population was not stratified by area given that spatial scale of the study site was small and aerial sampling techniques were used to determine their population estimates

1. Corresponding author, email: [b.bett@cgiar.org](mailto:b.bett@cgiar.org), phone: 254-20-4223453 [↑](#footnote-ref-1)
